# Antibody Responses to BNT162b2 Vaccination in Japan: Monitoring Vaccine Efficacy by Measuring IgG Antibodies against the Receptor-Binding Domain of SARS-CoV-2

**DOI:** 10.1128/spectrum.01181-21

**Published:** 2022-01-19

**Authors:** Hidetsugu Fujigaki, Yasuko Yamamoto, Takenao Koseki, Sumi Banno, Tatsuya Ando, Hiroyasu Ito, Takashi Fujita, Hiroyuki Naruse, Tadayoshi Hata, Saya Moriyama, Yoshimasa Takahashi, Tadaki Suzuki, Takahiro Murakami, Yukihiro Yoshida, Yo Yagura, Takayoshi Oyamada, Masao Takemura, Masashi Kondo, Mitsunaga Iwata, Kuniaki Saito

**Affiliations:** a Department of Advanced Diagnostic System Development, Fujita Health University Graduate School of Health Sciences, Toyoake, Aichi, Japan; b Department of Clinical Pharmacy, Fujita Health University School of Medicine, Toyoake, Aichi, Japan; c Center for Clinical Trial and Research Support, Fujita Health University School of Medicine, Toyoake, Aichi, Japan; d Department of Joint Research Laboratory of Clinical Medicine, Fujita Health University School of Medicine, Toyoake, Aichi, Japan; e Department of Clinical Laboratory, Fujita Health University Hospital, Toyoake, Aichi, Japan; f Department of Medical Laboratory Science, Fujita Health University Graduate School of Health Sciences, Toyoake, Aichi, Japan; g Research Center for Drug and Vaccine Development, National Institute of Infectious Diseases, Tokyo, Japan; h Department of Pathology, National Institute of Infectious Diseases, Tokyo, Japan; i Diagnostics Research Laboratories, Diagnostics Technical Service & Research Operations, Diagnostics Division, FUJIFILM Wako Pure Chemical Corporation, Amagasaki, Hyogo, Japan; j In Vitro Diagnostics Division, FUJIFILM Corporation, Tokyo, Japan; k Department of Respiratory Medicine, Fujita Health University School of Medicine, Toyoake, Aichi, Japan; l Department of Emergency and General Internal Medicine, Fujita Health University School of Medicine, Toyoake, Aichi, Japan; University of Mississippi Medical Center

**Keywords:** BNT162b2, COVID-19, SARS-CoV-2, serological assay, vaccine

## Abstract

To fight severe acute respiratory syndrome coronavirus 2 (SARS-CoV-2), which causes coronavirus disease 2019 (COVID-19), mass vaccination has begun in many countries. To investigate the usefulness of a serological assay to predict vaccine efficacy, we analyzed the levels of IgG, IgM, and IgA against the receptor-binding domain (RBD) of SARS-CoV-2 in the sera from BNT162b2 vaccinated individuals in Japan. This study included 219 individuals who received two doses of BNT162b2. The levels of IgG, IgM, and IgA against RBD were measured by enzyme-linked immunosorbent assay before and after the first and second vaccination, respectively. The relationship between antibody levels and several factors, including age, gender, and hypertension were analyzed. Virus-neutralizing activity in sera was measured to determine the correlation with the levels of antibodies. A chemiluminescent enzyme immunoassay (CLEIA) method to measure IgG against RBD was developed and validated for the clinical setting. The levels of all antibody isotypes were increased after vaccination. Among them, RBD-IgG was dramatically increased after the second vaccination. The IgG levels in females were significantly higher than in males. There was a negative correlation between age and IgG levels in males. The IgG levels significantly correlated with the neutralizing activity. The CLEIA assay measuring IgG against RBD showed a reliable performance and a high correlation with neutralizing activity. Monitoring of IgG against RBD is a powerful tool to predict the efficacy of SARS-CoV-2 vaccination and provides useful information in considering a personalized vaccination strategy for COVID-19.

**IMPORTANCE** Mass vaccination campaigns using mRNA vaccines against SARS-CoV-2 have begun in many countries. Serological assays to detect antibody production may be a useful tool to monitor the efficacy of SARS-CoV-2 vaccination in individuals. Here, we reported the induction of antibody isotype responses after the first and second dose of the BNT162b2 vaccine in a well-defined cohort of employees in Japan. We also reported that age, gender, and hypertension are associated with differences in antibody response after vaccination. This study not only provides valuable information with respect to antibody responses after BNT162b2 vaccination in the Japanese population but also the usefulness of serological assays for monitoring vaccine efficacy in clinical laboratories to determine a personalized vaccination strategy for COVID-19.

## INTRODUCTION

Coronavirus disease 2019 (COVID-19) is caused by severe acute respiratory syndrome coronavirus 2 (SARS-CoV-2) infection (1). Since the initial outbreak in Wuhan, China in late 2019, a health emergency with social and economic disruptions has spread worldwide. Efforts to develop a vaccine against SARS-CoV-2 to control the global COVID-19 pandemic became a global effort and resulted in the emergency approval of several vaccines (2–6).

One of the first approved COVID-19 vaccines, BNT162b2 (Pfizer/BioNTech), has shown promising efficacy in clinical trials (7, 8). BNT162b2 is an mRNA vaccine that expresses the full prefusion spike (S) glycoprotein of SARS-CoV-2. A two-dose regimen of BNT162b2 was found to be safe with 95% efficacy in preventing symptomatic COVID-19 in persons 16 years of age or older (8). Following the authorization of COVID-19 vaccines for emergency use by the U.S. Food and Drug Administration on December 11th, 2020, a mass vaccination campaign began throughout the world.

Now that it has been demonstrated that the COVID-19 vaccines can induce a humoral response thereby protecting individuals from symptomatic COVID-19, several studies attempted to use serological assays to detect antibody production following COVID-19 vaccination (9–19). To monitor the efficacy of vaccines, antibody titer can be used to predict protection against SARS-CoV-2, which is already done as a routine laboratory testing for many viruses, such as measles morbillivirus, rubella virus, and hepatitis virus.

There is a consensus that serological assays help predict vaccine efficacy; however, a variety of antigens used to detect antibodies and a variety of antibody isotypes measured in these assays may cause issues with interpreting the results (20, 21). In addition, to monitor the efficacy of a vaccine by measuring antibodies against SARS-CoV-2 in the clinical laboratory, it is necessary to measure antibodies that are highly correlated with serum neutralizing activity. A previous study from our group determined the kinetics and neutralizing activity of various antigen-specific antibody isotypes against SARS-CoV-2 in the serum of patients with COVID-19 (22). This study used enzyme-linked immunosorbent assay (ELISA) to measure IgG, IgM, and IgA for various antigens, including the full-length S, S1, receptor-binding domain (RBD), and nucleocapsid (N) proteins. As a result, the measurement of IgG against the RBD (RBD-IgG) not only showed good clinical performance for diagnosing SARS-CoV-2 infection but also exhibited a high correlation with serum neutralizing activity. Therefore, monitoring RBD-IgG may be the best indicator for quantifying the immunogenicity of vaccines.

To evaluate the usefulness of measuring anti-SARS-CoV-2 antibodies to monitor vaccination efficacy, we analyzed antibody responses, including RBD-IgG, -IgM, and -IgA after the first and second dose of the BNT162b2 vaccine in a well-defined cohort of employees in Japan. We also analyzed how the antibody response changes in correlation with several factors such as age, gender, and hypertension. Furthermore, we examined which antibody isotypes against RBD were strongly correlated with the neutralization titer. Finally, we developed and validated a chemiluminescent enzyme immunoassay (CLEIA) method to measure RBD-IgG for clinical settings. This study not only provides valuable information with respect to antibody responses after BNT162b2 vaccination in the Japanese population but also the usefulness of RBD-IgG for monitoring vaccine efficacy in clinical laboratories.

## RESULTS

**FIG 1:**
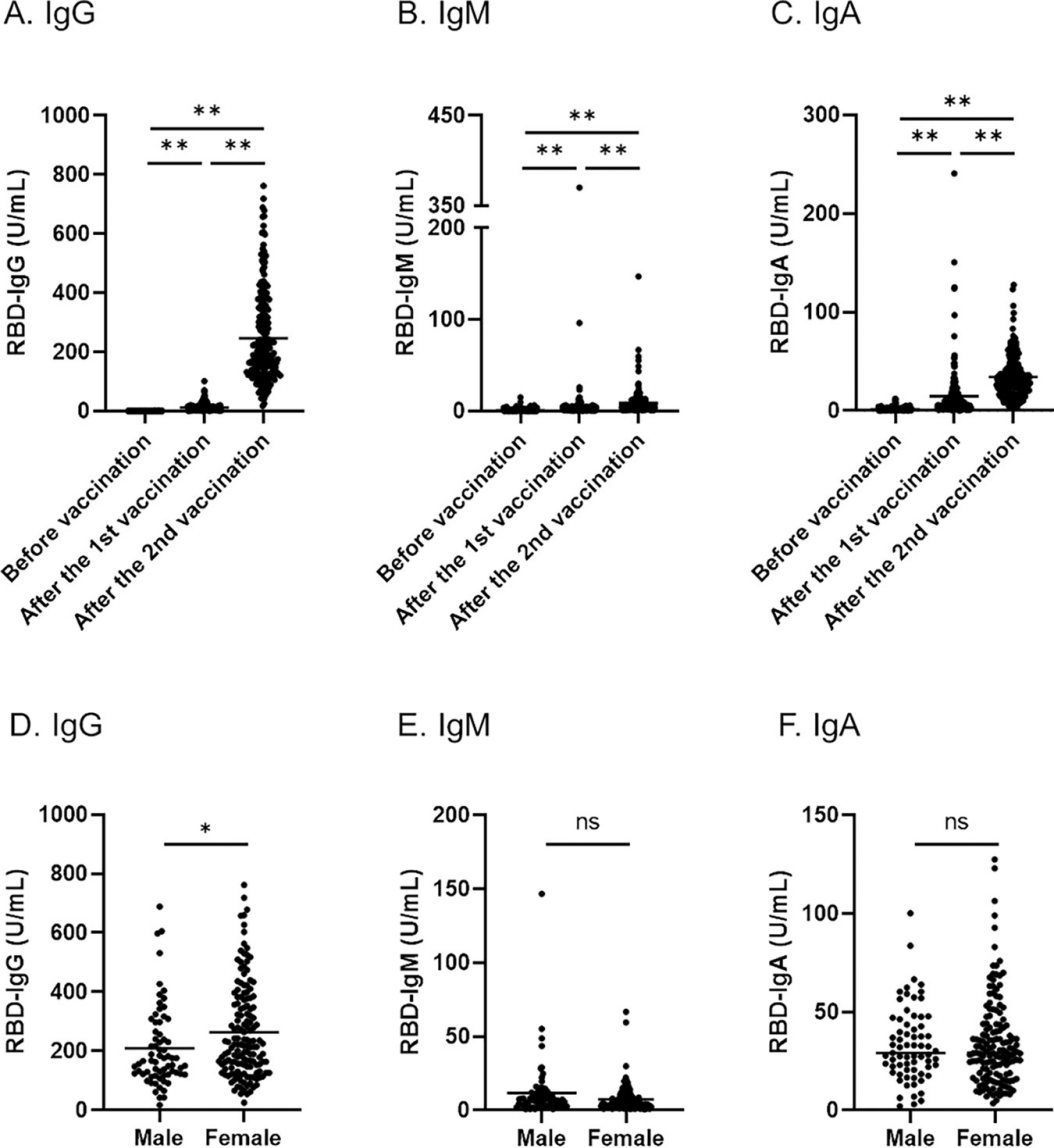
Antibody responses of IgG (A), IgM (B), and IgA (C) in all the subjects induced by BNT162b2 vaccination and differences of IgG (D), IgM (E), and IgA (F) levels between males and females after the second vaccination. Serum samples were collected before vaccination, after the first vaccination, and after the second vaccination (A to C). Antibody levels after the second vaccination were compared between males and females (D to F). The horizontal solid lines indicate the mean of antibody levels. Statistical analysis was done by a two-tailed Mann–Whitney test (*P < 0.05, **P < 0.01, ns: not significant).

Volunteers (*n* = 219) that participated in this study were vaccinated with two doses of the BNT162b2 mRNA vaccine. The characteristics of the subjects are shown in Table 1. Serum was collected 3 times according to the following time points: before the first vaccination, after the first vaccination, and after the second vaccination. The antibody titer of RBD-IgG, IgM, and IgA was measured by ELISA. All antibody titer increased after the first and second vaccinations compared with before the first vaccination but dramatically increased after the second vaccination (Fig. 1). The IgG titers in the serum of all subjects ranged between 0.0 and 1.8 U/mL with a mean of 0.1 ± 0.2 U/mL before the first vaccination, between 0.0 and 100.9 U/mL with a mean of 11.8 ± 12.8 U/mL after the first vaccination, and between 17.2 and 762.4 U/mL with a mean of 246.6 ± 153.6 U/mL after the second vaccination (Fig. 1A). The average titer after the second vaccination increased more than 20 times from the average titer after the first vaccination (*P* < 0.01). In serum after the second vaccination, IgM titers in all subjects ranged between 0.7 and 146.8 U/mL with a mean of 8.7 ± 13.1 U/mL and IgA titers ranged between 2.1 and 127.5 U/mL with a mean of 34.1 ± 21.5 U/mL (Fig. 1B and C).

**TABLE 1:** Characteristics of subjects in this cohort

Category	Gender	Group	No.	Age
		Total subjects	219	45.6 ± 11.0
Gender		Male	69	47.2 ± 12.3
		Female	150	44.8 ± 10.3
Age	Male	20-39yr	18	31.6 ± 6.3
		40-49yr	18	43.5 ± 3.2
		50-59yr	20	53.8 ± 3.0
		> 60 yr	13	63.8 ± 4.3
	Female	20-39yr	43	31.6 ± 5.6
		40-49yr	55	45.1 ± 2.7
		50-59yr	43	54.2 ± 2.6
		> 60 yr	9	61.3 ± 1.7
Hypertension		Male	12	59.1 ± 5.0
		Female	6	52.2 ± 5.3
Allergies		Male	35	45.8 ± 13.8
		Allergen (multiple answers)		
		Pollen	25	
		Animal	2	
		House dust	1	
		Unknown	8	
		Female	87	44.1 ± 10.9
		Allergen (multiple answers)		
		Pollen	64	
		Food	12	
		House dust	8	
		Animal	4	
		Drug	4	
		Alcohol	3	
		Others^*a*^	3	
		Unknown	8	

a Include sun, metal, and latex.

**FIG 2:**
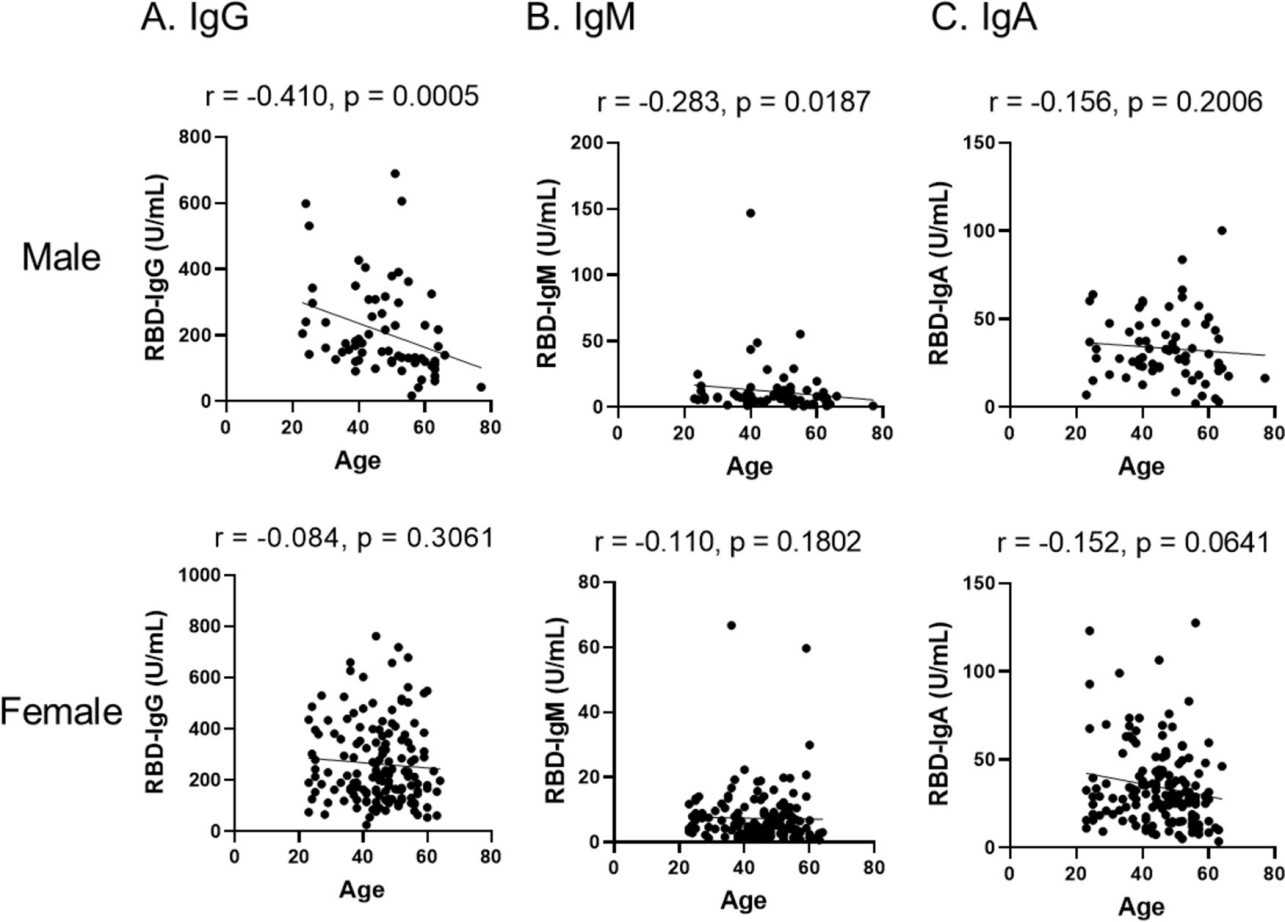
Correlation between age and IgG (A), IgM (B), and IgA (C) levels of males (upper panel) and females (lower panel) after the second vaccination. Correlation analysis was calculated using Spearman’s correlation coefficient. The values of Spearman’s r and p are presented in each figure.

The antibody titers showed differences between genders. Each antibody titer was compared between males and females. The IgG titer after the second vaccination of females was significantly higher than that of males (*P* < 0.05) (Fig. 1D). The average antibody titer was 263.6 ± 158.0 U/mL for females and 209.9 ± 137.6 U/mL for males. Previous data showed that age was also an important factor of antibody titer after vaccination (9). Therefore, correlations were established between age and each antibody titer. In males, age correlates negatively with RBD-IgG (r = −0.410) and IgM (r = −0.283) (Fig. 2A and B). In contrast, each antibody titer did not show a correlation with age for females (Fig. 2A and B).

**FIG 3:**
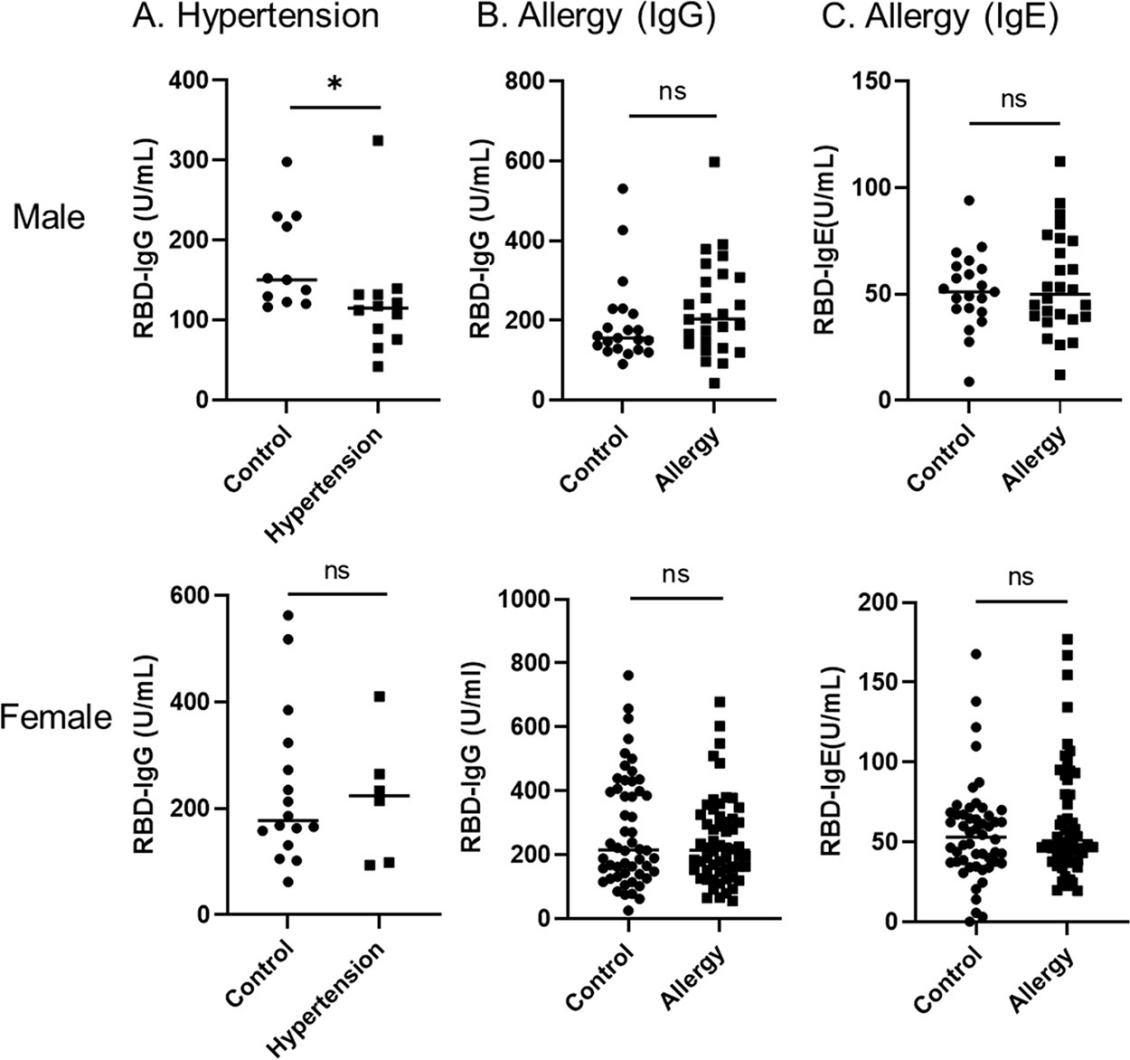
Levels of RBD-IgG in subjects who have hypertension (A) and levels of RBD-IgG (B) and RBD-IgE (C) in subjects who have an allergy. (A) Subjects diagnosed with hypertension with or without antihypertensive medications (12 males and 6 females) were selected as the hypertension group and compared to the age-matched control group (11 males and 16 females). (B and C) Subjects who have allergies (26 males and 58 females) were selected as the allergy group and compared to the age-matched control group (21 males and 52 females). The horizontal solid lines indicate the mean of antibody levels. Statistical analysis was done by a two-tailed Mann–Whitney test (*P < 0.05, ns: not significant).

Several diseases, such as cancer, chronic kidney disease, hypertension, and diabetes, have been reported to be associated with the severity of COVID-19. The IgG titers of hypertensive subjects were significantly decreased compared to control subjects in males, but there was no difference in females (Fig. 3A). Although more than 50% of participants had allergies in this cohort, the IgG titer did not show differences between those with allergies and controls (Fig. 3B). To elucidate whether allergy-affected the antibody titers, we measured IgE antibody against RBD using ELISA. After the second vaccination, serum IgE titer in all subjects ranged between 0.3 and 234.9 U/mL with a mean of 55.3 ± 31.3 U/mL. There was no difference in the amount of IgE between the allergy and control groups (Fig. 3C).

**FIG 4:**
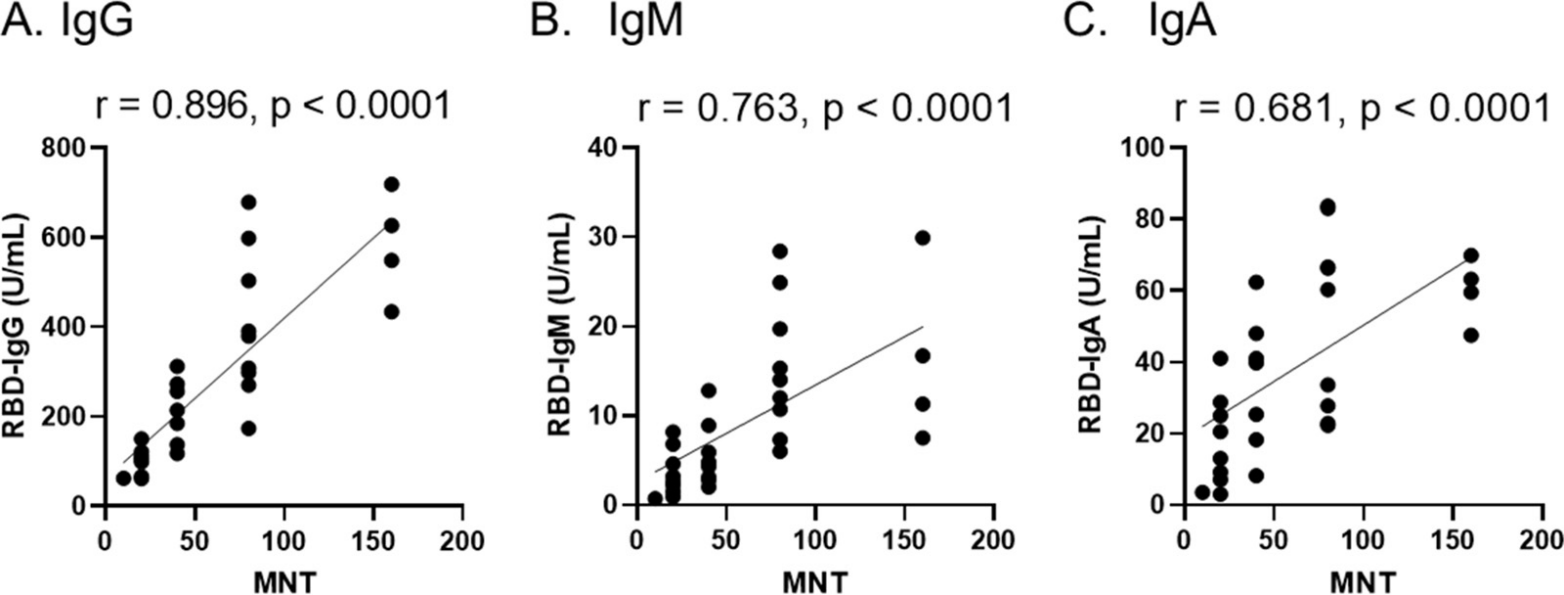
Correlation between virus-neutralizing activity and IgG (A), IgM (B), and IgA (C) levels after the second vaccination. Antibody measurement by ELISA and neutralization assays were performed using sera from 30 subjects after the second vaccination. The index of the highest sera dilution factor with cytopathic effect inhibition was defined as the microneutralization test titer (MNT). The correlation was calculated using Spearman’s correlation coefficient. The values of Spearman’s r and p are presented in each figure.

For protection against COVID-19 infection, whether the antibody exhibits neutralizing activity produced by vaccination is the most important factor. Among 219 serum samples collected from subjects after the second vaccination, 30 samples were selected considering the distribution of antibody levels, and virus-neutralizing activity was assessed. The correlation between antibody titer of IgG, IgM, and IgA and virus-neutralizing activity was examined in the serum of subjects after the second vaccination. Neutralizing activity correlated positively with IgG, IgM, and IgA titer (Fig. 4). Among the isotypes, IgG showed a higher correlation with neutralizing activity (*r* = 0.896).

IgG titers against the N-terminal domain (NTD) of SARS-CoV-2 have been reported to be involved in both enhancement and suppression of infection (23–25). Therefore, NTD-IgG titers were measured by ELISA in all subjects. The correlation between RBD-IgG and NTD-IgG was examined in the serum of subjects after the second vaccination. Antibody titer of RBD-IgG and NTD-IgG correlated positively (*r* = 0.755; Fig. S1A). Furthermore, NTD-IgG correlated positively with neutralizing activity (*r* = 0.789; Fig. S1B).

**FIG 5:**
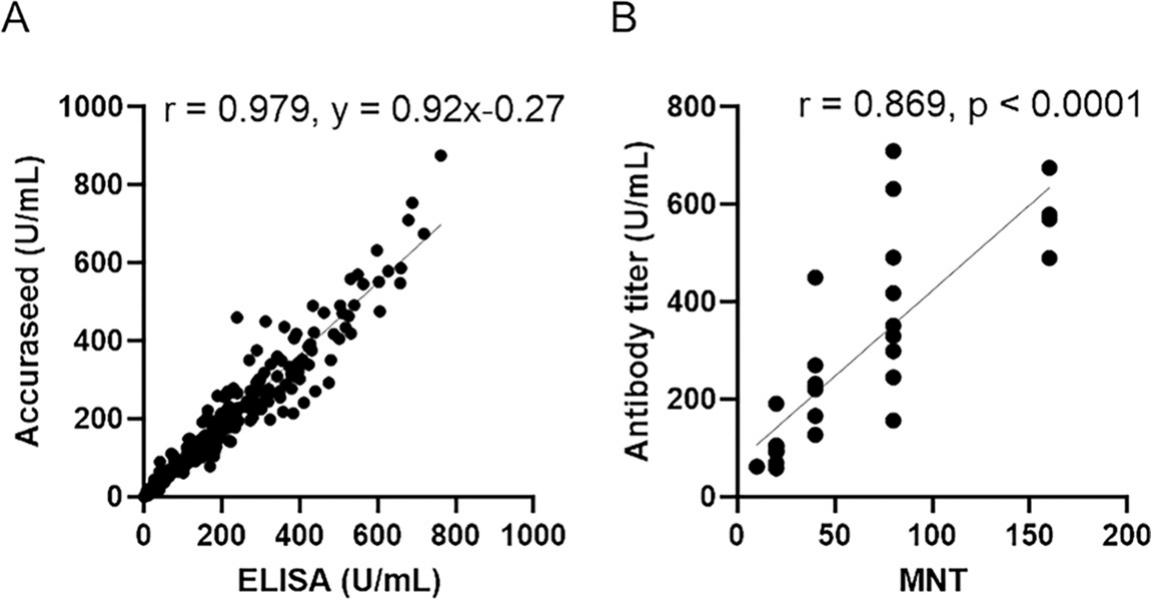
(A) Relationship between RBD-IgG levels measured by ELISA and CLEIA assay. RBD-IgG levels in serum samples from all subjects (a total of 657 samples) were measured by both ELISA and Accuraseed. Correlation R was calculated by Pearson’s correlation. (B) Correlation between virus-neutralizing activity and RBD-IgG obtained by Accuraseed. Antibody measurement by Accuraseed and neutralization assays was performed using sera from 30 subjects after the second vaccination. The index of the highest sera dilution factor with cytopathic effect inhibition was defined as the microneutralization test titer (MNT). The correlation was calculated using Spearman’s correlation coefficient. The values of Spearman’s r and p are presented.

A rapid and accurate method to monitor antibody titers is a useful tool to investigate the efficacies of vaccination in clinical laboratories. Therefore, we developed a new CLEIA method using a two-step sandwich method to measure anti-SARS-CoV-2 antibodies with the Accuraseed automated immunoassay system. This method measured antibody titers within about 10 min (Fig. S2). To validate the CLEIA assay, the limit of blank, the limit of detection and limit of quantification, repeatability (intraday precision), and linearity within assay range were established (Fig. S3 to S5, Table S1 to S3). To assess this new method, we examined the relationship between RBD-IgG levels measured by ELISA and that of the CLEIA assay. The correlation was determined between RBD-IgG levels by ELISA and RBD-IgG levels by CLEIA assay (*R* = 0.979; y = 0.92x − 0.27; Fig. 5A). Furthermore, the levels of the RBD-IgG by CLEIA assay correlated with the virus-neutralizing activity (*r* = 0.869; Fig. 5B). Increasing evidence suggests that disease severity is positively correlated with higher antibodies, of which IgG subclasses exhibited a weakly negative correlation with viral load. We compared the RBD-IgG levels by CLEIA assay with virus-neutralizing activity in patients with COVID-19. RBD-IgG levels determined by CLEIA assay in patients with COVID-19 showed a good correlation with neutralizing activity (*r* = 0.723; Fig. S6). These data indicate that the CLEIA assay is a useful tool to validate the efficiency of vaccination and monitor the severity of COVID-19 in patients.

## DISCUSSION

This study investigated the antibody response produced by the SARS-CoV-2 vaccination in a well-defined cohort of employees in Japan. In all enrolled subjects, the levels of RBD-IgG, IgM, and IgA antibody increased after vaccination compared to before the first vaccination. The levels of RBD-IgG in serum after the second vaccination showed a higher correlation with the virus-neutralizing activity compared with that of the other isotypes. A previous study that used COVID-19 patient serum showed that the levels of RBD-IgG were useful markers for diagnosing SARS-CoV-2 infection and neutralizing activity (22). These data indicate that the second dose vaccination potentiates neutralizing activity against COVID-19.

Persons who have been infected with SARS-CoV-2 are thought to have protective immunity and memory responses (26). IgM antibodies against SARS-CoV-2 are the first isotype to be generated against the novel antigen and can be detected as early as day 3 post COVID-19 symptoms (27). IgA antibodies also play an important role in mucosal immunity. IgA is the most important immunoglobulin for fighting infectious pathogens in the respiratory and digestive systems at the point of pathogen entry. As an immune barrier, secretory IgA can neutralize SARS-CoV-2 before it reaches and binds to epithelial cells (27). The present data show that the antibody titer of IgG, IgM, and IgA significantly increased after the second vaccination compared with before the first vaccination (Fig. 1). In some subjects, the levels of IgA or IgM antibody increased immediately after the first vaccination and decreased after the second vaccination (Fig. 1B and C). Further studies are needed to monitor the effects of isotype differences during SARS-CoV-2 infection, but our data suggest that two doses of vaccine are valuable to protect from the infection of SARS-CoV-2 because the titer of all isotypes increased in all subjects after the second dose of vaccine.

The RBD-IgG levels after the second vaccination of males were significantly lower than those of females (Fig. 1D). Several studies suggest that there are many differences between males and females with respect to the immune response to SARS-CoV-2 infection and inflammatory diseases (28). Females generally produce higher levels of antibodies which remain in the circulation longer. The activation of immune cells is higher in females than males, and it correlates with the trigger of TLR7 and the production of interferon. Therefore, women with COVID-19 show a better outcome than men (29). In addition, a previous study demonstrated that antibody responses of greater magnitude were shown in women versus in men after the second dose of BNT162b2 (9). These studies support our findings that females have a higher antibody-producing ability than males following the vaccination.

In the present study, elderly males had a lower RBD-IgG antibody-producing ability than their younger counterparts (Fig. 2A). Previous data has shown that there are age-related differences in antibody responses raised after BNT162b2 vaccination with lower frequencies of neutralizing antibodies in the elderly group (12). These data suggest that the vaccination is effective for all ages, but it is important to continue preventive measures for COVID-19, especially in elderly males at least until more longitudinal real-world data emerges.

Several diseases, such as cancer, chronic kidney disease, hypertension, and diabetes, have been reported to be associated with the severity of COVID-19. The present results show that the antibody titers of hypertensive subjects in males were significantly lower than control subjects, but there was no statistical difference in females. In addition, our analysis showed that there was no correlation between antibody titer and allergies. In this cohort, more than 50% of participants had allergies. Fifty years ago, allergies were almost unknown in Japan, but the number of patients has been increasing every year. Today, reportedly one out of every two people has an allergy (30). To elucidate whether allergy-affected antibody titers in this cohort, we measured IgE antibody against RBD using ELISA. Our data showed neither presence nor absence of allergy-affected IgE and IgG titers against RBD of SARS-CoV-2 (Fig. 3B and C). A previous study using a cohort of 248 healthcare workers in Italy reported that age and gender were statistically associated with differences in antibody response after vaccination, which is consistent with our results, whereas body mass index and hypertension had no statistically significant association (9). Further studies are needed to investigate what factors influence antibody responses from SARS-CoV-2 vaccination using a larger cohort.

For protection against SARS-CoV-2 infection, the most important factor is whether the antibody exhibits neutralizing activity produced by vaccination. Our data showed that among the antibody isotypes, IgG showed a high correlation with neutralizing activity. However, the effectiveness of antibodies against NTD remains controversial. Some reports have shown that antibodies against NTD protect against SARS-CoV-2 infection (24, 25), whereas others have shown that they enhance viral infection by changing the attachment of the virus to cells (23). Therefore, we measured IgG against NTD to investigate whether NTD-IgG correlates with RBD-IgG. Our results showed that antibody titers of RBD-IgG and NTD-IgG correlated positively (*r* = 0.755; Fig. S1A). Furthermore, NTD-IgG correlated positively with neutralizing activity (*r* = 0.789; Fig. S1B). Further research is needed, but the results of our study suggest that NTD-IgG seems to prevent viral infection.

We developed and validated a new method for measuring RBD-IgG using an automated CLEIA method. RBD-IgG measured by Accuraseed is a rapid, accurate, and precise method that strongly correlates with neutralizing activity (Fig. 5, Fig. S2 to S6, Table S1 to S3). Because monitoring antibody responses that correlate with neutralizing activity after SARS-CoV-2 vaccination is highly advisable for predicting protection against SARS-CoV-2, this method may become a useful tool to monitor the efficacy of vaccination in clinical laboratories.

In conclusion, this study demonstrated that IgG, IgM, and IgA antibodies against RBD were produced after BNT162b2 vaccination in all subjects. Among these antibodies, RBD-IgG correlated best with neutralizing activity. These results suggest that monitoring of RBD-IgG is a powerful tool to predict the efficacy of SARS-CoV-2 vaccination. The level of RBD-IgG after the second vaccination was affected by gender and age, which provides useful information to consider personalized vaccination strategies for COVID-19.

## MATERIALS AND METHODS

### Ethics statement, participants, and sample processing.

This study was approved by the Ethics Committee for Clinical Research of the Center for Research Promotion and Support at Fujita Health University (authorization number HM20-526 and HM21-167). The study was conducted in accordance with the accordance to Declaration of Helsinki. All participants provided written informed consent before undergoing any study procedure.

The study included a series of 219 employees at Fujita Health University, who received two doses of BNT162b2 at Fujita Health University Hospital. The demographic and clinical characteristics of the participants are presented in Table 1. No participant had a history of SARS-CoV-2 infection. Blood samples were collected from all participants 3 times: (i) before the first vaccination, (ii) an average ± SD of 14.6 ± 1.7 days after the first vaccination, and (iii) an average ± SD of 14.3 ± 1.6 days after the second vaccination. Serum was obtained by centrifugation for 15 min at 1,500 *g* at room temperature, aliquoted, and stored at −80°C until use.

To compare hypertension and control groups, we selected 45 samples (18 subjects with hypertension and 27 age-matched controls) from this cohort. The subjects in the hypertension group were selected according to the following criterion: subjects diagnosed with hypertension, regardless of antihypertensive medications used, and those in the control group were selected if they had no current use of medications. To compare the allergy and control groups, we selected 157 samples (84 subjects with allergy and 73 age-matched controls) from this cohort. The subjects in the allergy group were selected according to the following criterion: subjects diagnosed with allergy regardless of anti-allergy medications use, and those in the control group were selected according to the following criterion: no current use of medications.

For correlation analysis between antibody titers and neutralization activity using COVID-19 patient sera, we used 53 residual serum samples from 38 patients who were admitted to Fujita Health University Hospital from 28 February 2020 to 16 September 2020.

### Measurement of RBD-IgG, IgM, IgA, and IgE, and NTD-IgG by ELISA.

RBD-IgG was measured using an ELISA kit from FUJIFILM Wako Pure Chemical Corporation. All procedures were performed in accordance with the manufacturer’s instructions. IgM and IgA in serum samples were measured using ELISAs that were developed in the previous study (22). Briefly, 96-well plates (Thermo Fisher Scientific) were coated with 250 ng of recombinant RBD protein or NTD (Acro Biosystems) and used for ELISAs. Fifty microliters of each serum sample, which were diluted 1:10 for IgE, 1:201 for IgG and IgA, or 1:2010 for IgM assays in PBS containing 2% bovine serum albumin, were added per well and incubated at room temperature for 60 min. The plates were washed three times with PBS containing 0.1% Tween 20 (PBS-T), peroxidase-labeled anti-human IgM, IgA (Midrand Bioproducts), or IgE antibody (Abcam) was added as secondary antibody and incubated at room temperature for 60 min. After incubation, the plates were washed five times with PBS-T, and substrate solution (TMB/H_2_O_2_) (FUJIFILM Wako Pure Chemical) was added and incubated at room temperature for 10 min. The reaction was stopped by the addition of 1 M HCl, and the absorbance was measured at 450 nm using a 620 nm reference filter using a microplate reader (Molecular Devices). Serially diluted pooled serum from 10 patients with COVID-19 who had strong IgA and IgE positivity and 5 patients who had strong IgM positivity was used to prepare a standard curve. The OD_450_ values of the standard of 0.015 were converted to 1 U/mL and these values were fitted onto a line graph using linear regression analysis. The antibody units in the test serum samples were then calculated from their OD_450_ values using the parameters estimated from the standard curve.

### SARS-CoV-2 neutralizing assay.

The neutralizing assay was conducted according to the method described in a previous report with minor modifications (22, 31, 32). Briefly, serum samples were heat-inactivated at 56°C for 30 min and then serially diluted with Dulbecco’s Modified Eagle medium (FUJIFILM Wako Pure Chemical) supplemented with 2% fetal bovine serum (Biowest), 100 U/mL penicillin, and 100 mg/mL streptomycin (Thermo Fisher Scientific). The mixture of diluted sera (2-fold serial dilutions starting at 1:5 dilution) and 100 tissue culture infectious dose 50 hCoV-19/Japan/TY-WK-521/2020 strain (EPI_ISL_408667) was incubated at 37°C and 5% CO_2_, then placed on VeroE6/TMPRSS2 cells (JCRB1819, JCRB Cell Bank) seeded in 96-well, flat-bottom plates at 1 × 10^4^ cells per well and cultured at 37°C and 5% CO_2_. On day 5, the plates were fixed with 20% formalin (FUJIFILM Wako Pure Chemical) and stained with crystal violet solution (Sigma-Aldrich) to evaluate the cytopathic effect. The index of the highest serum dilution factor with cytopathic effect inhibition was defined as the microneutralization test titer (MNT).

### Measurement of RBD-IgG by CLEIA.

We utilized another laboratory test kit (33) for the Accuraseed automated immunoassay system (FUJIFILM Wako Pure Chemical Corporation) to develop a CLEIA method to measure RBD-IgG in serum samples. The Accuraseed is an automated immune analyzer based on a CLEIA method that combines antigen-antibody reaction and chemiluminescence reaction. This CLEIA method utilizes magnetic microparticles as a separating agent, which enables rapid measurement of RBD-IgG within about 10 min (34). Principles and procedures of RBD-IgG measurement using Accuraceed are shown in Fig. S2. A 10 μL serum sample and 140 μL of an immune reaction buffer were added to 12.5 μg of recombinant SARS-CoV-2 RBD-bound particles and incubated at 37°C for 3 min. After the bound and free fractions were separated, 50 μL of peroxidase-labeled anti-human IgG antibody was added and incubated at 37°C for 3 min, followed by separation of the bound and free fractions. Finally, 100 μL of the substrate solution and 100 μL of the hydrogen peroxide solution were added, and the amount of light emitted per unit time was measured. Using pooled serum from the patients with COVID-19 described above in the ELISA, a standard curve was generated, and the sample luminescence was applied to the calibration curve to calculate the RBD-IgG antibody units in the serum samples. The basic performance of the assay such as the limit of quantification, the limit of detection, accuracy, and precision are provided in the supplementary information (Fig. S3 to S5, Table S1 to S3).

### Statistical analysis.

Statistical analysis was performed using GraphPad Prism version 8.4.3 for Windows (GraphPad Software). Quantitative data were analyzed by a two-tailed Mann–Whitney U test and *P* < 0.05 was considered statistically significant. Correlation analysis was performed using Spearman’s correlation test. Linear regression was performed to assess the correlation of RBD-IgG levels between Accuraseed and ELISA.
