# Supplementary material for: Antibody Responses to BNT162b2 Vaccination in Japan: Monitoring Vaccine Efficacy by Measuring IgG Antibodies against the Receptor-Binding Domain of SARS-CoV-2
Source: Microbiol Spectr. 2022 Jan 19;10(1):e01181-21. doi: 10.1128/spectrum.01181-21 (PMC8768797; doi:10.1128/spectrum.01181-21)
Supplement: SUPPLEMENTAL FILE 1 — Supplemental material. Download SPECTRUM01181-21_Supp_1_seq10.pdf, PDF file, 0.3 MB [file spectrum01181-21_supp_1_seq10.pdf]

Figure S1.

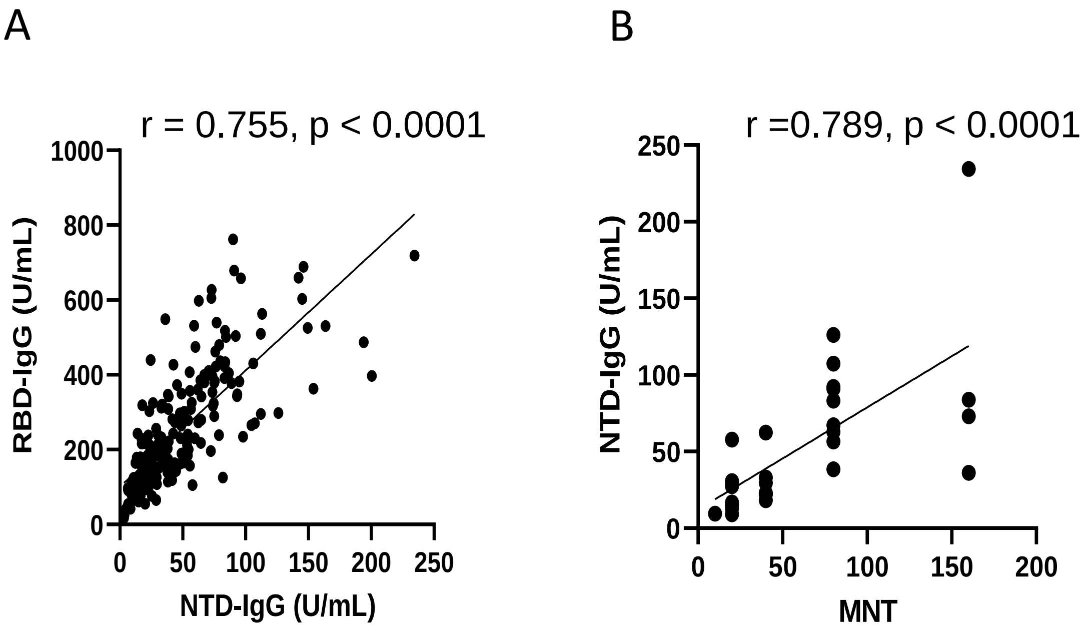

Fig. S1. Relationship between NTD-IgG levels and RBD-IgG levels, and correlation between NTD-IgG levels and virus neutralizing activity.
(A) NTD-IgG levels in serum samples after the second vaccination were measured by ELISA. Correlation r were calculated by Pearson's correlation. (B) NTD-IgG levels and neutralizing activity were measured using serum samples from 30 subjects after the second vaccination. The index of the highest sera dilution factor with cytopathic effect inhibition was defined as the microneutralization test titer (MNT). Correlation was calculated using Spearman's correlation coefficient. The values of r and p are presented.

Figure S2.

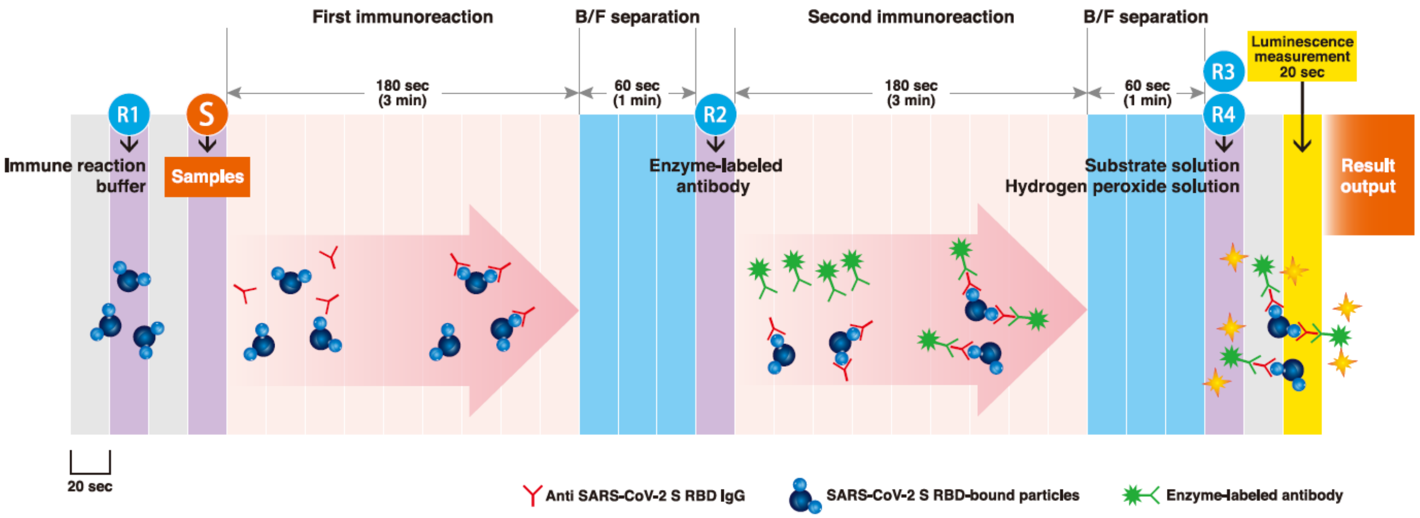

Fig. S2. Schematic diagram of principle and procedure of IgG measurement by Accuraceed using chemiluminescent enzyme immunoassay (CLEIA).

Figure S3.

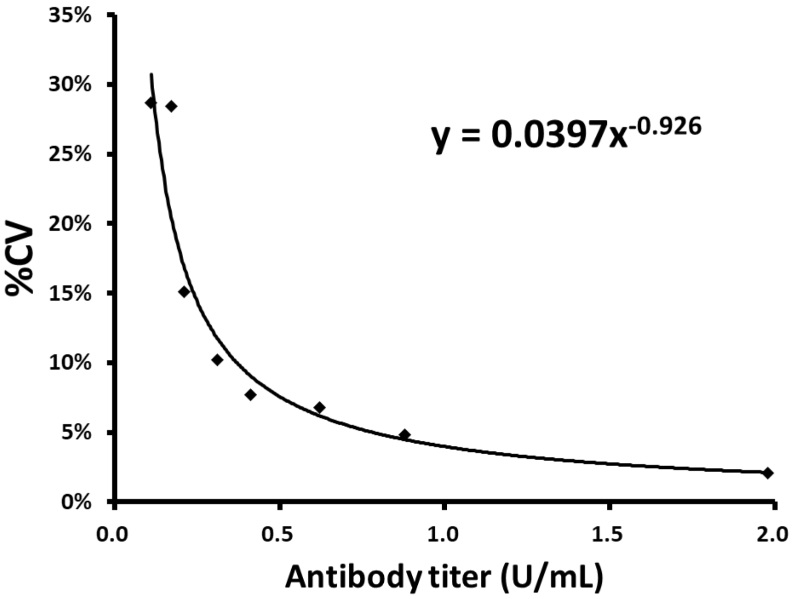

Fig. S3. Limit of quantitation of the RBD-IgG by CLEIA assay.
The limit of blank (LoB), limit of detection (LoD) and limit of quantification (LoQ) at coefficient of variation (CV) values of 10% were examined. These values were calculated by referring to EP17-P of the National Committee for Clinical Laboratory Standards (NCCLS).

Figure S4.

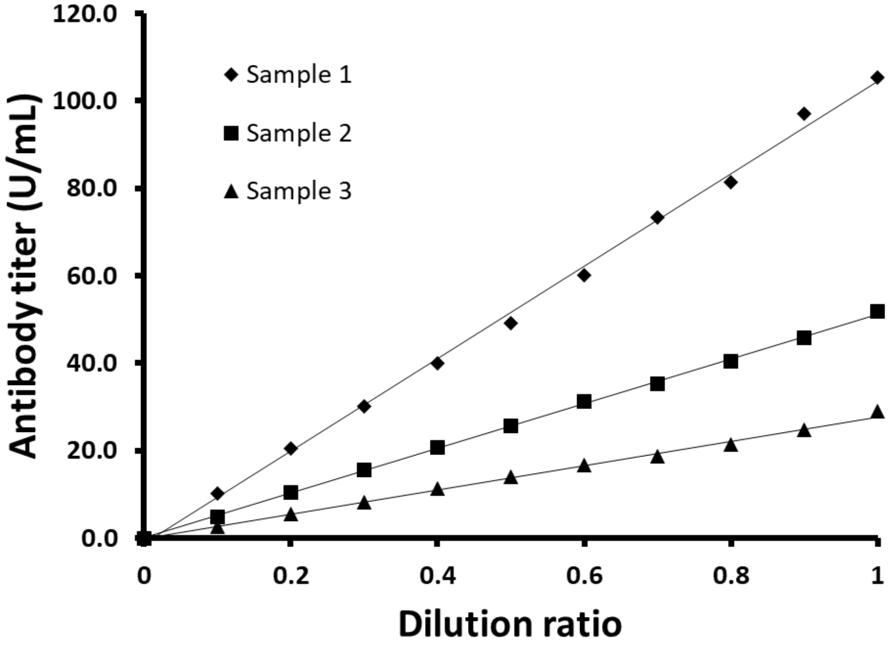

Fig. S4. The linearity of antibody titer measured by CLEIA assay.
Three serum samples, from low to high concentration, were diluted 10 times with diluent solution. Linearity was established in the range from 0 to 105.3 U/mL.

Figure S5.

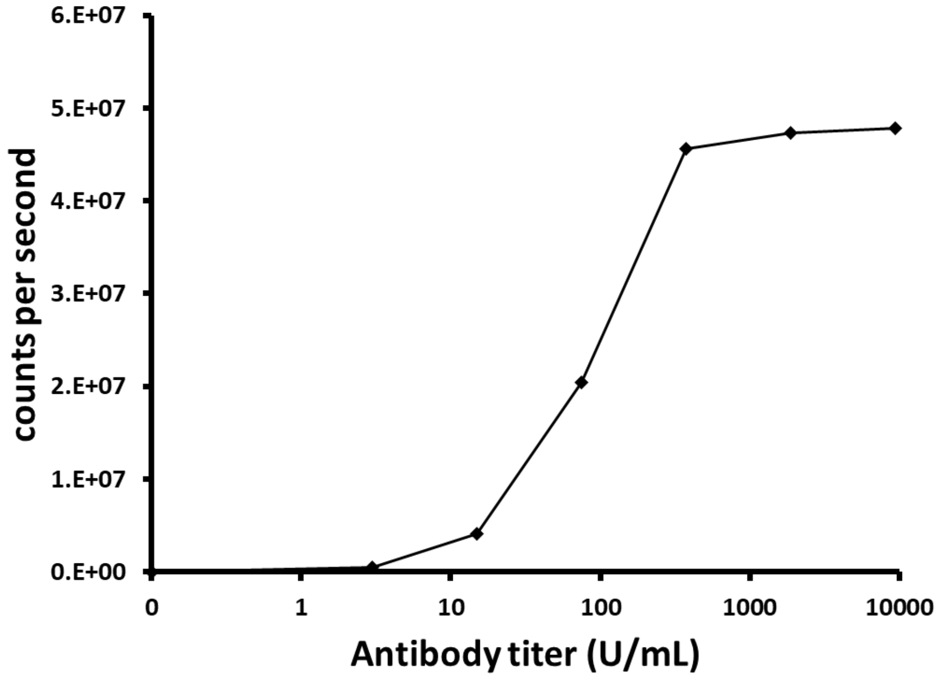

Fig. S5. Hook effect of antibody titer measured by CLEIA assay.
Serum sample was diluted from 3.0 to 9375.0 U/mL with diluent solution to evaluate the hook effect. The hook effect was not observed at concentrations up to 9375.0 U/mL.

Figure S6.

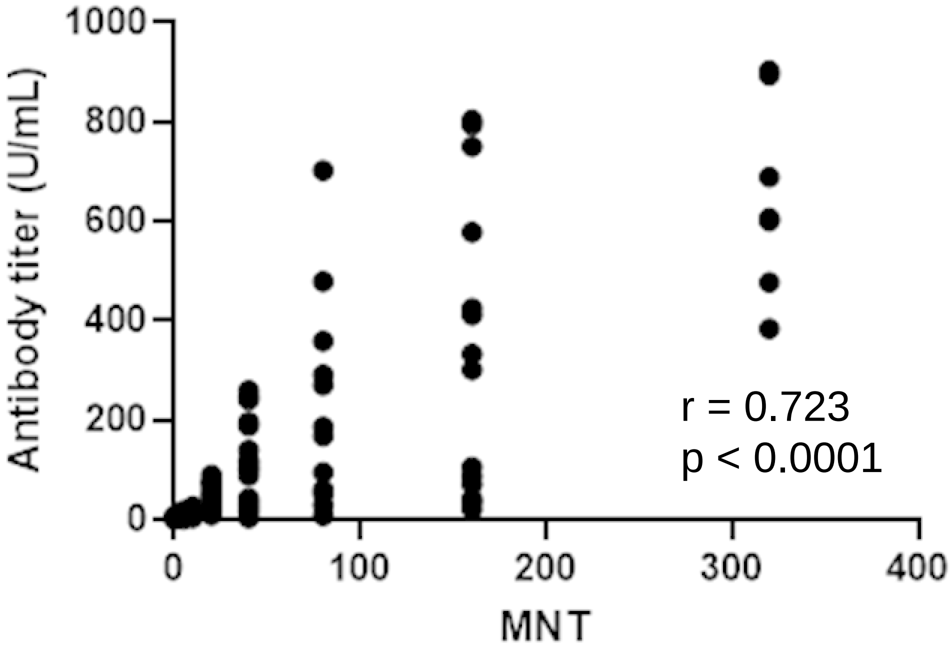

Fig. S6. Correlation between virus neutralizing activity and RBD-IgG levels in serum samples from patients with COVID-19.

Antibody titer and neutralizing activity were measured using 79 residual serum samples from 37 patients with COVID-19. The index of the highest sera dilution factor with cytopathic effect inhibition was defined as the microneutralization test titer (MNT). Correlation was calculated using Spearman's correlation coefficient. The values of Spearman's r and p are presented.

Supplemental Tables

Supplemental Table S1. Limit of blank (LoB), limit of detection (LoD), and limit of quantitation (LoQ) of the RBD-IgG CLEIA assay.

	Antibody titer (U/mL)
LoB	0.0
LoD	0.1
LoQ	0.4

Supplemental Table S2. Repeatability of the antibody measurements in serum samples using the RBD-IgG CLEIA assay. Data (mean and SD) are presented in U/mL.

	Sample 1	Sample 2	Sample 3
N	20	20	20
Mean	5.6	42.3	100
SD	0.1	1.6	2.7
CV	1.8%	3.8%	2.7%

Supplemental Table S3. Linearity of antibody units measured in serum samples using the RBD-IgG CLEIA assay.

Dilution		Serum 1	Serum 2	Serum 3
Antibody titer (U/mL)	0/0	0.0	0.0	0.0
	1/10	10.1	4.9	2.7
	2/10	20.6	10.5	5.6
	3/10	30.1	15.5	8.3
	4/10	40.0	20.7	11.4
	5/10	49.1	25.7	13.9
	6/10	60.1	31.2	16.6
	7/10	73.4	35.2	18.7
	8/10	81.4	40.4	21.4
	9/10	97.0	45.7	24.7
	10/10	105.3	51.8	28.9
Recovery rate	1/10	95.9%	94.6%	93.4%
	2/10	97.8%	101.4%	96.9%
	3/10	95.3%	99.7%	95.7%
	4/10	95.0%	99.9%	98.6%
	5/10	93.3%	99.2%	96.2%
	6/10	95.1%	100.4%	95.7%
	7/10	99.6%	97.1%	92.4%
	8/10	96.6%	97.5%	92.6%
	9/10	102.4%	98.0%	95.0%
	10/10	100.0%	100.0%	100.0%
